# Epigenetics and Shared Molecular Processes in the Regeneration of Complex Structures

**DOI:** 10.1155/2016/6947395

**Published:** 2015-11-22

**Authors:** Labib Rouhana, Junichi Tasaki

**Affiliations:** Department of Biological Sciences, Wright State University, 3640 Colonel Glenn Highway, Dayton, OH 45435-0001, USA

## Abstract

The ability to regenerate complex structures is broadly represented in both plant and animal kingdoms. Although regenerative abilities vary significantly amongst metazoans, cumulative studies have identified cellular events that are broadly observed during regenerative events. For example, structural damage is recognized and wound healing initiated upon injury, which is followed by programmed cell death in the vicinity of damaged tissue and a burst in proliferation of progenitor cells. Sustained proliferation and localization of progenitor cells to site of injury give rise to an assembly of differentiating cells known as the regeneration blastema, which fosters the development of new tissue. Finally, preexisting tissue rearranges and integrates with newly differentiated cells to restore proportionality and function. While heterogeneity exists in the basic processes displayed during regenerative events in different species—most notably the cellular source contributing to formation of new tissue—activation of conserved molecular pathways is imperative for proper regulation of cells during regeneration. Perhaps the most fundamental of such molecular processes entails chromatin rearrangements, which prime large changes in gene expression required for differentiation and/or dedifferentiation of progenitor cells. This review provides an overview of known contributions to regenerative processes by noncoding RNAs and chromatin-modifying enzymes involved in epigenetic regulation.

## 1. Introduction

**Figure 1:**
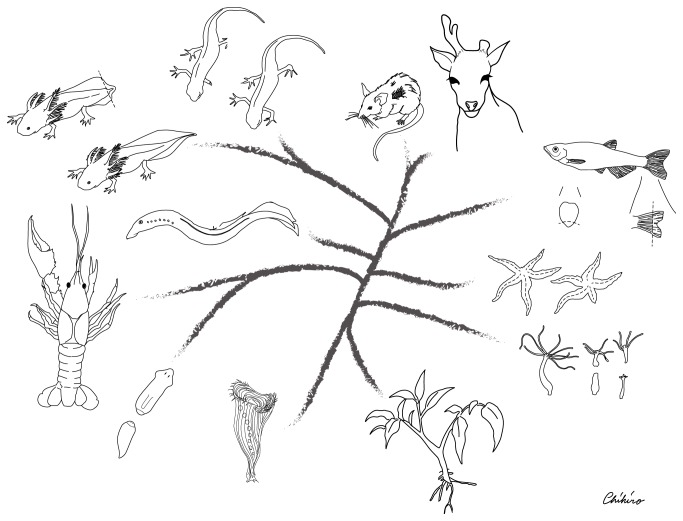
Phylogenetic distribution of regenerative organisms. Regenerative abilities tend to decline as complexity increases through evolution. For instance,* Hydra* and planarians can regenerate their whole bodies, whereas regeneration in deer or African spiny mice is limited to certain parts of their body such as antlers or skin, respectably. The following representatives from different phyla are illustrated: plants,* Stentor* (Ciliophora),* Hydra* (Cnidaria), planarian (Platyhelminthes), crayfish (Crustacea), starfish (Echinodermata), lamprey, fish, axolotl, and newt (Urodela), as well as deer and spiny mouse (Mammalia). Phylogenetic distances and organisms are not drawn to scale. Illustration contributed by Chihiro Uchiyama Tasaki.

Aristotle was captivated by the observation that lizards were capable of regrowing a tail after having it cut [1]. Regeneration—the ability to redevelop lost body parts—has been displayed in myths and folktales for centuries. Today, accumulating evidence shows that regenerative events that may seem fictitious are a reality in a wide range of organisms, from unicellular ciliates to large plants and animals (Figure 1). The regenerative capacities of different organisms vary immensely, as some are restricted to specific tissues or periods of time during development (e.g., the* Xenopus* tadpole tail), while others are capable of regenerating their entirety over uncountable occasions (e.g., planarian flatworms) [2, 3]. The mechanisms involved in regeneration have mystified observers throughout history and left them wondering whether a cellular permit forgiving the loss of a limb or an eye could be uncovered and shared with us, the unlucky humans who seem obligated to get through life with only one set of body parts.

Over 300 years ago, the famous French entomologist René-Antoine Ferchault de Réaumur reported detailed observations of crayfish claw regeneration [4]. Réaumur's detailed accounting of the regenerative process is often credited for creating awareness about this topic amongst the scientific community. Since, descriptions of regeneration events in vertebrates have been reported widely, ranging from limbs, tails, and retinas of Urodele amphibians (i.e., newts and salamanders) [5–10] to hearts and fins of fish [11, 12], deer antlers [13], and skin of spiny mice [14]. The analysis of cellular and molecular mechanisms involved in natural regenerative phenomena is of great interest to improve medical applications for replacement of lost or damaged tissue in humans.

## 2. Mechanistic Similarities of Regeneration Processes

Even though the study of vertebrates and crustaceans has uncovered regenerative capabilities that surpass the expectations of past and present scientists, their capacity for regeneration remains relatively modest when compared to a collection of invertebrates that rely (at least partially) on asexual reproduction. Freshwater organisms belonging to the genus* Hydra* (named after the mythological multi-headed monster futilely decapitated by Hercules) can reproduce asexually through “budding,” which involves the development and detachment of an individual from somatic tissue of the “parent.” Similarly, planarian flatworms can reproduce asexually through “fission,” which involves separation of a tail piece from the body of the “parent” followed by regeneration of missing structures by both anterior and posterior fragments. These organisms are not only able to develop their entire anatomy from somatic tissue during asexual reproduction but also capable of regenerating their entire body from a small piece of tissue upon injury. Slicing a planarian into 20 different fragments could result in the formation of 20 completely functional descendents. Early reports describing the regenerative potential of these organisms [15, 16] were followed by decades of experimental investigation based on amputations, dissections, transplantations, and microscopic analyses. Ultimately, these studies were the foundation of current investigations using modern molecular techniques to identify the genes and cellular processes that drive regeneration [17–19]. The revival of regeneration research in the era of molecular genomics, RNA-interference, and modern microscopy has resulted in detailed experimental accounts of the regenerative processes in a wide range of organisms. Altogether, these studies have illustrated fundamental mechanistic commonalities and differences involved in regeneration of complex structures; a few of these are detailed below.

### 2.1. Distalization Followed by Intercalation

Agata et al. (2007) proposed that a common phenomenon shared amongst complex regenerative events, be it a newt limb or the entire head of a polyp or a planarian, was the initiation of regenerative deployment by establishment of the most distal structure first (distalization) followed by a subsequent expansion of the structures in between (intercalation) [20]. This view contrasts with previous models in which the regenerative process was thought to take place as a progression from proximal to distal, akin to mason laying bricks to build a wall. Normally, complex tissue regeneration establishes the identity of the furthest end of the missing tissue and gradually develops the regions in between. Although perplexing at first sight, distalization and intercalation seem logical if one considers that embryonic development constitutes a continuously morphing and moving mass of cells that follow signaling gradients and not a linear progression from one end to the organism to the other. Regeneration does not reinvent development; it applies preexisting mechanisms utilized during embryogenesis.

### 2.2. Programmed Cell Death and Cellular Proliferation

Analyses of the initial events that follow tissue loss and wound healing in flies, planarians, frogs, and mice have revealed that signals released by dying cells induce a proliferative response in progenitor cells of regenerating tissue [21]. There are two major modes by which cells die: “necrosis,” which occurs when cells are exposed to unusual conditions or ruptured, and “apoptosis,” in which the cell actively participates in its own demise. It is still unclear whether necrotic cells that arise from tissue damage release any molecules that specifically induce downstream regenerative events. On the other hand, studies in varied regenerative contexts support that a burst in apoptosis occurs following tissue amputation [21]. Apoptotic cells near the wound site release signaling factors (e.g., Wnt3 in* Hydra*; [22]) that induce the increased proliferation of progenitor cells that are needed to support the redevelopment of missing tissue. Apoptosis also plays a role in later steps of the regenerative process, during which preexisting tissue rearrangement guides the functional connection and proportionality of new and old parts of the organism [23].

### 2.3. The Futile Search for the “Regeneration Gene”

It may seem unsatisfactory to propose that cellular events that occur during regeneration are not exclusive to this phenomenon. Wound healing is a common process that occurs in regenerative and nonregenerative tissue. Growing limbs, retinas, or heads are events that take place during normal embryonic development. The surprise is that so many organisms are capable of replicating embryonic processes as adults by reactivating developmental genetic pathways within the context of differentiated, previously grown tissue. So what then is the secret to regeneration? One key component is the availability of proliferative cells with the potential to differentiate into the cellular makeup of the missing tissue, whether these are obtained from reservoirs of stem cells or reactivation and reprogramming of differentiated cells in response to injury (see below). At the same time, a wealthy accumulation of stem cells does not guarantee that regeneration. This concept is beautifully demonstrated recently in studies of planarians with decreased regenerative capabilities from three different continents [24–26]. These studies showed that the evolutionary loss of head regeneration observed in some planarian species was not due to insufficient populations of stem cells, but by differences in expression levels of components in the conserved Wnt/*β*-catenin developmental pathway. The influence of the Wnt/*β*-catenin pathway on regeneration is not unique to invertebrates such as planarians and hydra; this pathway also controls digit regeneration in mice [27]. Another developmental signaling circuit that can dictate mammalian digit regeneration outcomes is the Noggin/Bone Morphogenic Protein (BMP) pathway. Yu et al. (2010) demonstrated that Noggin inhibits capable digit regeneration, whereas the fate of nonregenerating amputation wounds was reversed by BMP treatments that reinitiate digit tip development at the wound [28]. The identity of the signals driving regeneration after wound healing may vary throughout evolution, as long as activating proliferation and differentiation of progenitors in the correct spatiotemporal context is achieved.

### 2.4. “Stemness” and Cellular Sources for Regeneration

The presence of regenerative abilities in a wide range of organisms distributed throughout the animal kingdom suggests the evolutionary conservation of mechanisms involved in regeneration [29, 30]. A difference that has become apparent amongst the mechanisms that drive regeneration in different organisms is the source of cells used when redevelopment of missing tissue is needed. “Simpler” organisms such as* Hydra*, planarians, acoels, sponges, and plants rely on reservoirs of somatic stem cells classified as pluripotent, or highly multipotent, which continuously proliferate and differentiate to provide for regeneration, growth, and homeostatic maintenance. On the other hand, regenerative events in more complex organisms, such as regeneration of a vertebrate limb, heart, or retina, depend on cells with limited lineage differentiation potential, which often arise from dormant or dedifferentiated cells [31]. Humans continuously repair their intestinal epithelium through use of small reservoirs of intestinal stem cells that continuously proliferate and differentiate into a handful of epithelial cell types [32]. These cells, however, would be expected to fail at restoring damaged tissue in the heart or kidney, due to their limited potency. On the other hand, amphibian limb regeneration does not rely on reservoirs of stem cells, but rather on the partial reprogramming and proliferation of cells with restricted identities (e.g., muscle cells come from muscle) [33]. Surprisingly, the cell-type within each tissue that serves as the source for specific tissues during limb regeneration varies even in closely related species of salamanders [34]. These observations suggest that regenerative constraints are not established by the absence of stem cells or a specific cell type and that there are different possible avenues to achieve regeneration of complex structures.

In summary, regeneration of complex structures depends on the ability of cells to undergo significant changes in proliferative activity followed by activation of specific differentiation programs. In some cases, proliferation and differentiation are preceded by partial trans- or dedifferentiation. Each of these processes requires large changes in gene expression profiles, which could be facilitated by extensive changes in chromatin structure. Consequently, one would predict that epigenetic regulators have broad—and possibly conserved—contributions to regenerative phenomena. The remainder of this short review describes recent evidence for involvement of epigenetic regulation during regenerative events observed in different phyla.

## 3. Observations of Epigenetic Regulation in Regenerative Processes

Much effort has been invested in describing the contributions of epigenetic regulators to maintenance and differentiation of stem cells* in vitro*. Regeneration is the ultimate stage to analyze the molecular mechanisms that regulate adult cell “stemness” and differentiation. Recent evidence describing the contributions of three major modes of epigenetic regulators to regenerative phenomena is described below.

### 3.1. DNA Methylation

An abundant epigenetic tag in plant and vertebrate genomes is the methylated form of the DNA base cytosine known as 5-methylcytosine. This modification occurs within CpG dinucleotide repeats and has a role in silencing gene expression by blocking access of transcription factors and other proteins to DNA [35]. Although detection of this epigenetic mark has been negligible in the genomes of yeast, nematode, fly, and flatworm model systems [36, 37], it is abundant in algae, moss, plants, and vertebrates, as well as in* Ciona*, honeybees, and beetles [36, 38, 39]. The enzymes responsible for deposition of methyl groups on the C-5 position of cytosine are known as DNA methyltransferases (DNMTs). Homologs of DNMT3 are responsible for establishment of* de novo* DNA methylation patterns, whereas DNMT1 homologs reiterate such molecular arrangements overtime [40].

Plants retain profound regenerative abilities that have proven advantageous not only to survive damage in their natural habitats but also in their propagation from “cuttings” by horticulturists, as well as production of whole plants from cultured transgenic cells in biotechnology and agriculture. Initial observations of epigenetic regulation during plant regeneration came from studies of a crown gall tumor line derived from Transfer DNA (T-DNA) delivery through Agrobacterium infection of tobacco plants [41]. Phenotypic variation was observed in clonal cell lines expressing different T-DNA transcripts, some of which appeared normal due to silenced T-DNA that was heavily methylated. More recently, it has been established that differences in cytosine methylation distribution are not limited to T-DNA but extend throughout the genome of plants regenerated from* in vitro* culture systems [42]. Additionally, changes in methylation and activity of corresponding loci have been reported in response to physical stress [43]. Although many of the methylated loci correspond to silenced transposable elements, there is clear evidence for regulation of cellular pathways crucial to the regenerative potential of plants via cytosine methylation. Such is the role of MET1, a DNA methyltransferase that modulates expression of factors involved in transduction of auxin signaling. This hormone is largely responsible for the regulation of plant regenerative potential [44].

Mammalian cells lines can be reprogrammed into stable induced pluripotent stem (iPS) cells through a process that involves gradual demethylation of important pluripotency loci [45, 46]. Incomplete loss of methylation during dedifferentiation contributes to somatic “memory” in iPS cells [47]. Interestingly, similar cellular “memory” is illustrated in various vertebrate regenerative events, such as the aforementioned example of axolotl limb amputation, where cells giving rise to the regenerating limb retain tissue identity (new muscle comes from old muscle cells; new skin comes from old skin cells) [33]. Thus, one would suspect that partial changes in DNA methylation patterns occur in dedifferentiating and differentiating cells during regeneration of specific tissues. Although comprehensive analyses of changes in DNA methylation signatures during vertebrate limb regeneration remain to be analyzed, it has been observed that activation of* shh* during limb regeneration (which is required for proper limb development) correlates with the DNA methylation status of a conserved enhancer region required for expression of* shh* in limbs [48]. Expression of* shh* during limb regeneration was found to correlate with methylation of the enhancer region known as Mammalian Fish Conserved Sequence 1 (MFCS1). Interestingly, MFCS1 was found to be hypermethylated in adult* Xenopus* limbs, when regenerative abilities are limited, but hypomethylated during developmental stages when full limb regeneration is possible [48]. Additionally, Yakushiji et al. (2007) showed that methylation of MFCS1 was low in adult limbs of amphibian species with high regenerative potential (i.e., axolotl and newt). Thus, it appears that the methylation status of crucial developmental genes may not only change to allow for regeneration, but, as this case suggests, it is rather the preestablished patterns of low methylation that may be crucial to allow flexibility and reactivation of important loci during regeneration. This idea is supported by studies in zebrafish, where Müller glia transition from quiescent supportive cells to progenitor cells for lens regeneration. It was observed that although genes encoding for DNA demethylation and methylation machinery are activated at early and later times during lens regeneration, respectively [49], it was also found that DNA methylation of promoters of genes encoding for important pluripotency factors is already low in quiescent Müller glia, suggesting a “primed” state for genes contributing to stemness during regeneration precursor cell reprogramming.

### 3.2. Histone Modification

The study of posttranslational modification of histone tails by methylation and acetylation may be the most general and extensive type of epigenetic regulation observed in eukaryotes. Regulation of gene expression through modification of histones is also tightly connected to other mechanisms of epigenetic regulation discussed in this review, since preceding DNA methylation and noncoding RNA targeting often result in silenced chromatin structures via histone methylation. Events influenced by factors involved in histone modifications have been identified in various regenerative contexts. Perhaps the earliest evidence for methylation of histones during regeneration came from observations during liver regeneration in rats, in which highest levels of histone methylation were observed after cellular proliferation in response to amputation and likely in the process of new cell differentiation [50, 51].

The catalytic component of the Polycomb Repressive Complex 2 is encoded by* enhancer of zeste 2* (EZH2) orthologs, a family of methyltransferases that modify histone 3 at position Lysine 27 (H3K27me3) and mark important developmental loci for transcriptional repression [52]. This form of histone methylation regulates the differentiation of embryonic and adult stem cells in a stepwise fashion [53, 54]. H3K27me3 levels have been shown to decrease during zebrafish fin regeneration leading to reactivation of loci important for the regenerative process [55]. Similarly, global studies of chromatin showed that H3K27me3 was the only histone modification differentially established between cells of the ventral and dorsal iris during newt lens regeneration [56]. This is interesting because removal of the lens leads to an initial regenerative response through transdifferentiation from both dorsal and ventral irises; however ventral cells cease in their response and only cells from the dorsal iris end up contributing to regeneration [57]. Maki et al. (2010) showed that H3K27me3 stayed constant in cells of the dorsal iris but increased in the ventral iris, suggesting that increased silencing through the Polycomb Repressive Complex may inhibit contributions of cells from the ventral iris to lens regeneration [56]. A negative effect on regeneration by increased H3K27me3 methylation, more directly EZH2 activity, was reported in muscle regeneration in mice [58]. It was shown that inflammation-activated signaling in muscle satellite stem cells lead to increased PRC2-mediated inhibition of* Pax7* expression, a gene whose function is required for stem cell proliferation during muscle regeneration [58]. Similarly, EZH2 inhibition also increased transdifferentiation during imaginal disc regeneration in flies [59] and wound healing in mice [60]. Altogether, several lines of research show that the flexibility of precursor cells to display “stemness” in regenerative processes of different organisms depends on maintaining low levels of H3K27 methylation or, alternatively, increasing the activity of H3K27me3 demethylases [55, 61].

Planarian flatworms have become a fruitful model for identification of molecular mechanisms that take place during regeneration. Given their exceptional ability to undergo whole-body regeneration, as well as the experimental amenability for high-throughput analyses of gene expression and function in these organisms [17–19], it has become possible to test the contributions of numerous molecular pathways to the process of regeneration. Planarian regeneration is fueled by a large population of adult pluripotent stem cells, which are present in the mesenchyme throughout the life of these organisms. A number of recent reports have begun to uncover how chromatin regulation contributes to adult stem cell driven regeneration of the planarian body. Detection of histone modifications using commercial antibodies raised against well-conserved sequence epitopes from other organisms indicated the presence of H3K9K14ac, H3K4me2, and H3K9me3 in planarian cells [62]. Surveys of epigenetic histone modification enzymes by sequence conservation have identified dozens of putative chromatin regulators in the genome of the widely utilized planarian* Schmidtea mediterranea* [62–64]. From the identified enzymes related to chromatin regulation, a histone deacetylase homolog (HDAC-1) has been shown to be required for planarian regeneration and stem cell maintenance [63, 65]. Planarian homologs of the SET1/MLL family of histone methyltransferases, which catalyze H3K4 methylation, as well as members of the associated COMPASS and COMPASS-like complexes, have also been characterized [63]. SET1/MLL family homologs are expressed in both the stem cell population and differentiated planarian tissues [63]. Most importantly, inhibition of* set1* homolog expression was shown to lead to stem cell depletion and loss of regeneration [63]. Given the experimental ability to separate planarian stem cells and differentiated cells by fluorescence-activated cell sorting (FACS) [66], it will be interesting to identify loci with different patterns of histone marks between these cell types and during regeneration. It will also be intriguing to analyze how tinkering with the function of chromatin regulators affects the distribution of epigenetic modifications in the planarian genome and whether there is any conservation with loci targeted for regulation during regeneration of other genomes.

### 3.3. Noncoding RNA

It has become evident from work fueled by transcriptomic sequencing that the presence of regulatory noncoding RNAs (ncRNAs) is extensive in both eukaryotic and prokaryotic genomes. Some of the pioneer studies that demonstrated the importance of ncRNAs in regulating gene expression came from the analysis of 21–25 nucleotide (nt) length molecules known as microRNAs (miRNAs) crucial to nematode development [67, 68]. Although miRNAs work mainly at the posttranscriptional level by base-pairing with sequence at the 3′UTR of target mRNAs and blocking their translation, their discovery fueled sequencing expeditions that uncovered other types of ncRNAs. Three classes of ncRNAs shown to direct epigenetic regulation of chromatin include endogenous small-interfering RNAs (which are of ~21 nt length and are mainly found in plants), long (>200 nt) noncoding RNAs (lncRNAs), and PIWI-interacting RNAs (piRNAs) [69–72]. Of these, lncRNAs and piRNAs have surfaced as regulators in complex tissue regeneration contexts.

The identification of lncRNAs has increased substantially due to advances in Next-Generation RNA sequencing technologies. Thousands of lncRNAs are expressed in mammalian genomes, some of which have been shown to have tissue-specific, temporal, or disease-specific distribution [73, 74]. Although characterization of lncRNA functions has just begun, a few are thought to affect regeneration of skeletal muscle through possible epigenetic mechanisms. The* Dppa2* Upstream Binding Muscle lncRNA (Dum) is expressed in skeletal myoblast cells and promotes damage-induced muscle regeneration [75]. Dum was shown to work by repressing expression of the pluripotency factor* Dppa2* through recruitment of DNMTs [75]. Another lncRNA, the Myo-D Upstream Noncoding (MUNC) lncRNA, was shown to be required for murine muscle regeneration [76]. MUNC is transcribed from a region upstream of the Myo-D gene, but it was proposed to regulate promoters of other genes through mechanism yet to be identified [76]. As stated above, the characterization of lncRNA functions has just begun and there is already a strong indication that this category of molecules will play important roles in regeneration.

The PIWI subfamily of proteins belongs to the ARGONAUTE/PIWI family and was first characterized for the ability of its members to silence transposable element gene expression in the germline of popular animal model organisms [77, 78]. PIWI proteins work in association with piRNAs (24–31 nt length) at the posttranscriptional level to destroy target mRNAs and also silence loci of homologous sequence through epigenetic mechanisms [77]. Although the vast majority of identified PIWI/piRNA targets contain transposon sequence, there are a few known genes with cellular and developmental functions regulated by this pathway [77, 79]. Interestingly, several groups have observed strong expression of PIWI homologs in stem and/or progenitor cells that contribute to regeneration of invertebrate species of exceptional regenerative capacities [80]. These include planarians [81–84], acoels [85], sponges [86], ctenophores [87], and hydra [88, 89]. These observations suggest that expression of PIWI homologs may be a characteristic shared with ancestral somatic stem cells [80, 90]. Given that genomic integrity of adult somatic stem cells is paramount to the homeostatic maintenance and asexual reproduction of many of these organisms, one would expect that expression of PIWI homologs is required to serve that function. However, several lines of evidence suggest that the function of the PIWI/piRNA pathway goes beyond protecting the genomic integrity of stem cells from transposable events. First, planarian flatworms subjected to PIWI homolog* smedwi-2* RNAi fail to regenerate regardless of stem cell availability, which suggests that SMEDWI-2 may play a role in differentiation [84]. Secondly, some have estimated that only 20–30% of sequenced planarian piRNAs comprise transposable element sequence [91], which raises obvious questions regarding the function of the 70–80% of remaining piRNAs. Finally, activation of expression of PIWI homologs has been observed during regeneration of vertebrate limbs [92] and mammalian liver [93], which suggests that PIWIs have functions outside of stem cells during regeneration.

How does the PIWI/piRNA pathway influence the process of regeneration outside its role in maintaining genomic stability? One possibility is that important specific mRNAs must be destroyed by PIWIs during processes of differentiation. However, a more appealing possibility is that epigenetic mechanisms guided by piRNAs mediate extensive changes in chromatin structure necessary for cellular reprogramming during regeneration. piRNAs direct epigenetic silencing through recruitment of DNA methylation, H3K9 methylation, and/or direct interactions between PIWI and Heterochromatin Protein 1 (HP1) [77]. Since piRNAs target hundreds to thousands of loci for epigenetic silencing throughout the genome, their effect on chromatin covers not only transposable element sequence loci and direct target loci but also may extend to neighboring genes. This approach not only makes piRNAs the center of a major mechanism for epigenetic reprogramming as recently proposed [94], it suggests an active role for transposable element sequences as docks for regulation in activity of large chromosomal regions.

Planarian flatworms represent a system in which PIWI function during regeneration has been (and continues to be) extensively studied [81–84]. Although DNA methylation has failed to be detected in planarians [37], both H3K9 methylation and a requirement for HP1 function in stem cells of these organisms have been reported [62, 64]. Additionally, piRNAs and PIWI proteins have been shown to be associated with sequences representing transposable elements and genes with indispensable cellular functions, such as those coding for core histones [81, 91, 95]. Interestingly, genes and pseudogenes with homology to histone sequence are represented by hundreds of loci in the genome of the planarian* Schmidtea mediterranea* [62, 81], which is comparable to numbers of loci representing transposable element sequences. Expression of histone genes shuts down quickly in the process of planarian stem cell differentiation, so it is possible that piRNAs targeting these transcripts are activated to destroy histone mRNAs and silence respective loci for successful reprogramming during differentiation. This is one mechanism by which PIWI proteins and piRNAs could contribute to major changes in differentiation/reprogramming of cells during regeneration.

## 4. Closing Statements

Advances in genomic sequencing, manipulation, and stem cell biology have reinvigorated the study of regeneration. Now more than ever we are able to learn about the different ways in which a multitude of organisms overcome loss of tissue. The study of regeneration not only reveals the secrets of this fascinating phenomenon, but it also uncovers developmental pathways of differentiation, molecules that influence the longevity and memory of cells, as well as the control of cellular proliferation. Uncovering the function of machineries of epigenetic regulation in the context of regeneration will demonstrate how changes in chromatin drive differentiation and dedifferentiation of stem and progenitor cells* in vivo*.
